# Microscopic imbibition characterization of sandstone reservoirs and theoretical model optimization

**DOI:** 10.1038/s41598-021-87856-x

**Published:** 2021-04-19

**Authors:** Xuan Xu, Yujin Wan, Xizhe Li, Yong Hu, Shanshan Tian, Qingyan Mei, Chunyan Jiao, Changmin Guo

**Affiliations:** 1 PetroChina Research Institute of Petroleum Exploration and Development, Beijing, 100083 China; 2 China Petroleum Pipeline Engineering Corporation, Hebei, 065000 China; 3 Research Institute of Southwest Oil and Gas Field Company, Sichuan, 610051 China

**Keywords:** Hydrology, Energy science and technology, Physics

## Abstract

Traditional porous media imbibition models deviate from the actual imbibition process in oil and gas reservoirs. Experimental studies on gas–water imbibition in reservoirs were carried out to describe the dynamic profile variation process of wet phase saturation in reservoirs and to further reveal the variation of the imbibition front and the imbibition amount. Optimization and correction methods were established, and experimental verifications were performed. Studies have shown the following: (1) Unlike homogeneous porous media, the water phase imbibition process in oil and gas reservoirs is more complicated, and it is impossible for the maximum saturation of imbibition to reach 100%. (2) Contrary to the theoretical hypothesis, the imbibition of water is not piston-like, and there is a clear transition zone at the imbibition front. This transition zone is the main cause of water saturation variations in the imbibition zone; with the expansion of the imbibition zone, the influence of the transition zone on water saturation weakens. (3) Traditional theoretical models predict a positive correlation between the imbibition amount and the measurements; however, there is a large deviation in the numerical values, which must be corrected. (4) The L-W model was optimized and the parameter group fluid factor *F* and the reservoir factor *R* were proposed to characterize the properties of the fluid and the reservoir, respectively. These two parameters have a clear physical significance and are easy to accurately test. After experimental correction, the optimized model is favourably suitable for oil and gas reservoirs.

## Introduction

Imbibition refers to the process in which the wetting phase in porous media is sucked into the pores by the capillary force to replace the non-wetting phase. The displacement between oil, gas, and water caused by imbibition in oil and gas reservoirs plays an extremely important role in the accumulation and development of hydrocarbon. Oil recovery by imbibition represents an important mechanism for recovery ratio enhancement technologies such as fracturing stimulation and waterflood stimulation in oil reservoirs^1–4^. For gas reservoirs, the negative effect of water phase imbibition is more prominent; imbibition results in water intrusion at the edges and bottom of fractured gas reservoirs, while water phase trapping occurs in low-permeability tight gas reservoirs; these phenomena are important mechanisms that affect the recovery ratio of gas reservoirs. Along with the evolution of natural gas development technologies and hydraulic fracturing in recent years, the important role of water phase imbibition is more prominent in the water intrusion at the edges and bottom of fractured gas reservoirs and the exploration of tight gas and shale gas^5–9^. Therefore, the need for studies on the mechanism and theory of imbibition in oil and gas reservoirs is increasingly urgent^10–13^.

As a common natural phenomenon in porous media, imbibition has received continuous attention in various fields such as groundwater engineering, material science, mechanical engineering, aeronautics, and astronautics^14–18^. As early as the early twentieth century, Lucas and Washburn established the classic Lucas-Washburn (L-W) imbibition model for liquid wetting, which is used in many areas^19,20^. Then, in 1960, Handy developed the Handy model with water saturation in mind based on his assumption that the water imbibes in a piston-like manner^21^; the model was later improved by Li and Horne, et a1.^22,23^. Furthermore, Terzaghi derived a one-dimensional cylindrical soil self-imbibition model^24^, in consideration of the significant deviation from experiment results, including differences in the order of magnitude^25^; Lu and Likos further improved the model, however, their improved model involves difficult computations because it contains many parameters^26^. Mattax, Kyte, and other scholars proposed the oil and water imbibition scale equation (MK model) for fractured reservoirs and experimentally verified it under certain conditions^27^. However, Zhang et al. pointed out some errors in the experimental calculations^28^. With the development of fractal geometry theory in recent years, Cai et al. developed fractal models of spontaneous imbibition in porous media to improve the classic L-W imbibition law^29^; then, Li et al. performed fractal analysis to understand the effect of microscopic heterogeneity on imbibition^30^. Zhao systematically studied the L-W imbibition model systematically from the microscopic scale^31^. Li et al., and Du et al. carried out a study on imbibition theory of sandstone reservoir^32,33^. In general, the L-W model and the Handy model have had a more extensive and far-reaching impact compared to other conventional models. Theoretical research and model improvements based on these two models continue to this day^34–37^. Since the beginning of the twenty-first century, the petroleum sector has focused more on oil recovery by imbibition^38,39^. Chinese scholars started studying imbibition relatively recently. On an engineering application-oriented basis, their research findings have mostly focused on the mechanism of oil recovery by imbibition and improvements in the recovery ratio. As a result, there are relatively few theoretical studies on imbibition^40–42^. Generally, many mechanism problems and fundamental mechanics problems have not been completely solved due to the complexity of imbibition itself. Studies frequently introduce many assumptions or empirical constants while neglecting certain kinetic factors or over-simplifying theoretical models. As a result, theoretical calculations often do not fit experimental results well. Moreover, experimental results often vary among studies. Together, these limitations impose challenges to the applicability of theoretical models, especially their applicability and guiding role in complex porous media such as oil and gas reservoirs^43^.

To address the above-noted issues in previous studies on the mechanism and theory of imbibition, we combined theory and experiments in the present study. The summarization of the characterization of imbibition was achieved under the guidance of the theoretical analysis. The theoretical model of imbibition was verified and amended through experiments, thereby offering theoretical support for application of the theoretical model of imbibition in the accumulation and development of oil and gas, and the waterproofing and control of gas reservoirs.

## Mathematical theory

As stated earlier, the early and widely used theoretical models in the area of porous media imbibition include the traditional L-W imbibition model and the Handy model.

### L-W model

Vertical imbibition in porous media results from the synthetic actions of the capillary force, gravity, the viscous force, the inertial force related to the imbibition velocity, and other factors. The classical L-W equation is expressed as^19,20^

1 $${\uppi }r^{2} \rho gh + {\uppi }r^{2} \rho \frac{\partial }{\partial t}\left( {h\frac{dh}{{dt}}} \right) + 8{\uppi }\mu h\frac{dh}{{dt}} = 2{\uppi }r\sigma \cos \theta$$

where $r$ is average pore radius of porous medium, m. ${\uprho }$ is fluid density, ${\text{kg}}/{\text{m}}^{3}$. *g* is the gravitational acceleration, $g = 9.8{\text{ m}}/{\text{s}}^{2}$. *h* is height of imbibition front, m. $\sigma$ is interfacial tension, ${\text{N}}/{\text{m}}$. $\theta$ is contact angle, (°).$\mu$ is the fluid viscosity, ${\text{Pa}}$. *s*.*t*. is imbibition time, s.

From left to right, the equation consists of a gravity term, an inertial force term, a viscous force term, and a capillary force term. In imbibition, if the vertical distance is not large, the gravity can be ignored. Moreover, in view of the small inertial force of fluid flow during imbibition, Lucas and Washburn removed the inertial and gravity factors from Eq. (1) to obtain the following equation:

2 $$8{\uppi }\mu h\frac{dh}{{dt}} = 2{\uppi }r\sigma \cos \theta$$

The equation above is solved as

3 $$h{ = }\sqrt {\frac{{\sigma r{\text{cos}}\theta }}{2\mu }t}$$

According to the L-W hypothesis, imbibition is a uniform piston-like movement, so the porous media imbibition mass is given as

4 $$W_{L - w} = \rho A\emptyset h{ = }\rho A\emptyset \sqrt {\frac{{\sigma r{\text{cos}}\theta }}{2\mu }t}$$

where $A$ is contact area between porous media and fluid, ${\text{m}}^{{2}}$. $\emptyset$ is the porosity of the porous media, fraction.

Equations (3) and (4) are expressions of the imbibition height and amount, respectively, according to the L-W model.

First, the L-W model assumes that imbibition is a uniform piston-like process; second, the wet phase fluid will surely fill all pores in the case of imbibition; that is, the saturation is 100%. However, experiments have demonstrated that the imbibition in sandstone reservoirs involves a non-piston-like phenomenon^44^ and that it was difficult for wet phase saturation to reach 100% in reservoir during imbibition^21,22^. Model assumptions and simplifications will inevitably introduce errors. Moreover, the L-W model requires calculation of the mean hole radius of porous media. For homogeneous, regular porous media, the hole radius r is easy to determine; but when for oil and gas reservoirs with complex pore structures in which the pores and throats vary in size, the hole radius r is difficult to measure accurately. These problems have greatly restricted application of the L-W model for oil and gas reservoirs.

### Handy model

From the classical Darcy seepage theory, Handy assumed that water imbibes in a piston-like manner and established the Handy model of gas–water imbibition, considering water saturation but not taking into account gravity^21^:

5 $$h{ = }\sqrt {\frac{{2P_{c} K_{w} }}{{\emptyset S_{w} \mu_{w} }}t}$$

where $P_{c}$ is average capillary force, ${\text{Pa}}$. $K_{w}$ is water permeability, ${\text{m}}^{2}$. $S_{w}$ is water saturation of imbibition zone, fraction.

Because the Handy model assumes piston-like imbibition and defines the water saturation $S_{w}$ in the imbibition zone as a constant value,

6 $$W_{H} = \rho A\emptyset hS_{w}$$

Substituting Eq. (5) into Eq. (6) yields an expression for the imbibed water quantity, per the Handy model:

7 $$W_{H} = \rho A\sqrt {\frac{{2P_{c} K_{w} \emptyset S_{w} t}}{{\mu_{w} }}}$$

The Handy model exhibits a certain advancement by taking into account the effect of water saturation during imbibition. However, the assumptions that the water imbibes in a piston-like manner and the water saturation in the imbibition zone is a constant value are not essentially different from the assumptions of the L-W model. Therefore, the Handy model has the same problems as the L-W model. Furthermore, direct measurement would be difficult even if the water saturation $S_{w}$ in the imbibition zone is a constant value. Additionally, the capillary force $P_{c}$ and the water phase permeability $K_{w}$ are tightly associated with the water saturation $S_{w}$; a minor change in $S_{w}$ may bring about drastic changes in $P_{c}$ and $K_{w}$. These facts increase the uncertainty when applying the Handy model to the exploration and development of oil and gas reservoirs.

In conclusion, the hypotheses and simplification methods of the traditional L-W model and the Handy model are quite at odds with the actual imbibition process in oil and gas reservoirs, which will surely introduce many errors; the difficulty in accurately measuring the relevant parameters in the models further leads to their inapplicability in oil and gas reservoirs. Accordingly, it is essential to study relevant theories and experiments, to optimize and correct theoretical models based on a full understanding of the mechanism of imbibition, and to summarize the characterization of imbibition.

## Experiments and results

### Methodology

Previous imbibition experiments have typically determined the total imbibition amount and recovery ratio of reservoirs using the weight method, and the imbibition process in the reservoirs was seldom studied. As technologies have advanced, stratified NMR-based imaging equipment has been designed to detect signals from different parts of a core through stratified scanning, thereby yielding two-dimensional saturation images of score and revealing the imbibition dynamics of reservoirs^45,46^. The present experiment used the weight method with NMR technology to study gas–water imbibition in reservoirs.

A total of 12 rock specimens were taken from a sandstone reservoir for the imbibition experiment. The distribution range of the gas log permeability of the specimens was 0.022–3.104 mD, while the distribution range of the porosity was 4.79–15.29%. To ensure the contact area of gas was consistent with that of water, the core diameter was unified to 2.51 cm. See Table 1 for specific parameters.

**Table 1:** Basic parameters and partial experimental results of sandstone samples.

Sample number	Length, $L$ /cm	Porosity, $\emptyset$/%	Gas permeability, $K_{a}$/mD	Reservoir factor, $R$/($10^{ - 9} \cdot {\text{m}}^{5/2}$)	Water imbibed at the end of the experiment, $\Delta_{m}$/g	Water saturation at the end of the experiment, ${\text{ S}}_{{{\text{wmax}}}}$/%
S1	3.77	6.74	0.022	7.48	0.57	45.05
S2	3.81	4.79	0.024	5.94	0.54	60.16
S3	3.85	5.87	0.034	7.55	0.57	50.33
S4	3.87	6.63	0.105	11.05	0.73	57.99
S5	3.59	6.46	0.216	12.94	0.81	70.55
S6	5.82	10.74	0.402	22.10	1.89	61.24
S7	3.78	10.99	0.471	23.43	1.52	73.79
S8	3.85	9.80	0.505	21.89	1.41	75.91
S9	3.64	13.37	1.018	32.87	1.38	57.39
S10	3.64	15.29	1.123	37.28	1.41	51.33
S11	5.85	13.10	1.671	36.65	2.73	71.90
S12	5.76	13.62	3.104	43.99	2.95	76.00

**Figure 1:**
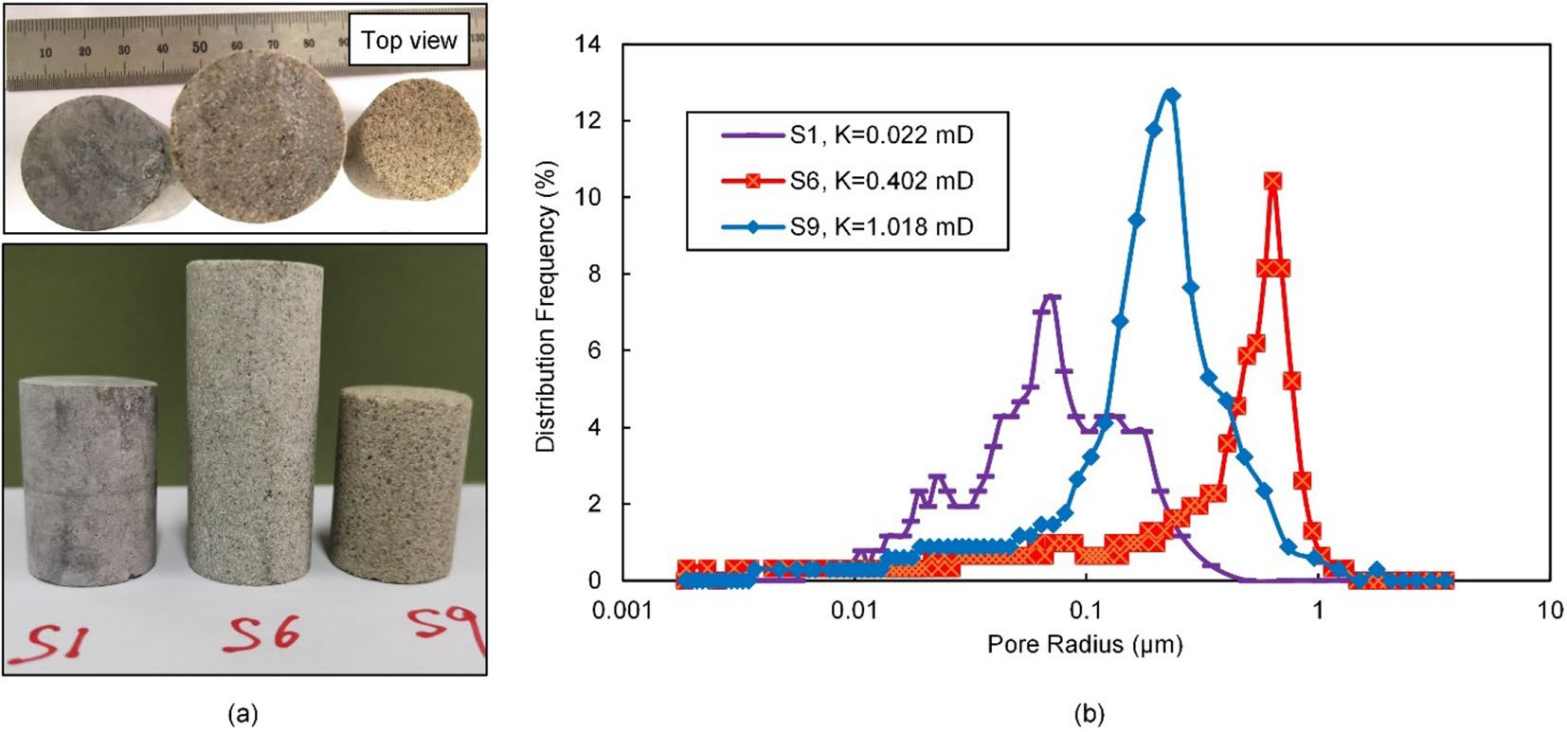
Experimental samples, (**a**) Photographs of samples S1, S6, and S9; (**b**) High-pressure mercury injection test results for samples.

Figure 1a shows photographs of three typical sandstone samples S1, S6, and S9. Figure 1b is the results of high-pressure mercury injection test for the three samples. The results show that the pore structure of sandstone is complex and has strong heterogeneity. The pores size and distribution frequency of different samples are different. For cores with greater permeability, the proportion of large pores are generally higher.

**Figure 2:**
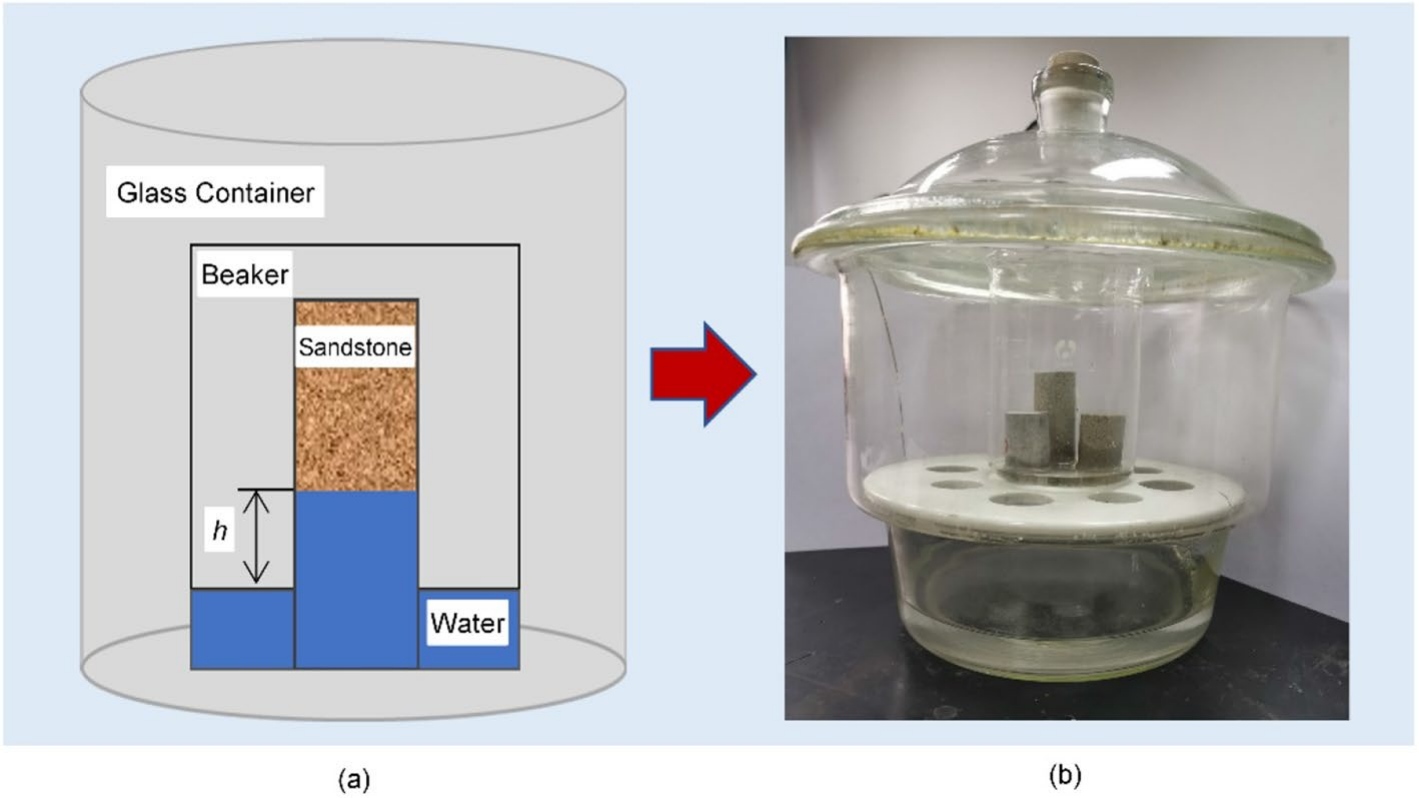
Schematic diagram and device picture for gas–water imbibition experiments.

Figure 2 shows the experimental schematic diagram and device picture for gas–water vertical imbibition. The experimental procedure is as follows:

1. Dry the specimen. After measuring the dry weight, place the specimen in a vacuum chamber, completely saturate it with water, and then measure the porosity φ.
2. Dry the specimen again, and conduct the imbibition experiment under normal temperature and pressure conditions. Detailed steps: Soak the lower end of the cores in a beaker of distilled water. To protect the water from evaporation, the beaker is placed in a glass container. Considering the consistency of the boundary conditions, immerse the lower end of the core 0.1 cm below the liquid level through calibration with a scale. Weigh the core regularly, and record the quantity of imbibed water.
3. As an example, during the imbibition experiment, typical specimen S6 with a permeability of 0.402 mD and a porosity of 10.74% was subjected to stratified NMR-based imaging tests for various imbibition durations to determine the water saturation profile and the location of the imbibition front in the reservoir during measurements of the imbibition of the specimen.
4. The experiment lasted for over 50 h; when the quality of the core became stable, which indicated that the imbibition in that specimen had essentially reached the maximum mass, the experiment was terminated.

### Variation of macroscopic water imbibition

**Figure 3:**
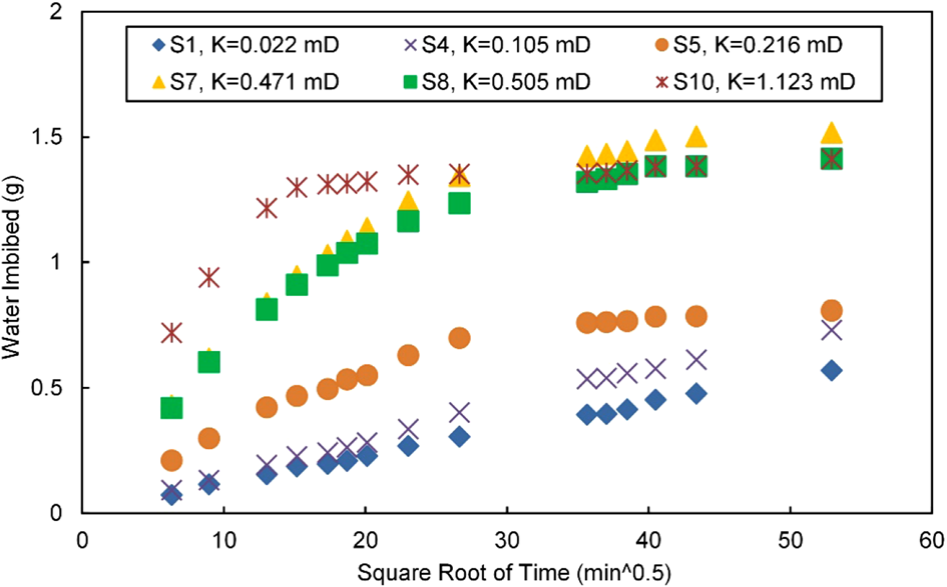
Water imbibition versus the square root of time.

Figure 3 shows the variation in the quantity of imbibed water in the 6 specimens over time. The theoretical analysis of imbibition according to the L-W model and the Handy model demonstrated that there was a strong positive linear correlation between the water imbibition and the square root of time in all specimens during the early stage of imbibition (230 min; 15.2, when converted to the square root of min). After 230 min, the curves successively began to dip away from the linear relationship, indicating that the water imbibition growth had slowed. Cores with higher porosity and permeability values, such as S8 and S10, exhibited faster deviations, and their imbibed water quantities more quickly approached a fixed value. The analysis results suggest that the imbibition front started to progressively approach or reach the upper edge of the specimen after 230 min; the higher the reservoir porosity and permeability values were, the more quickly the front rose, the sooner it reached the upper boundary, and the sooner the imbibition saturation tended to stabilize.

The variation processes of imbibition water saturation and the maximum imbibition saturation are key points in experimental studies on imbibition. The weight method is normally used to calculate the water saturation of the core as a whole:

8 $$S_{wt} = \frac{{\Delta_{m} }}{{\rho_{w} A\emptyset L}}$$

where $S_{wt}$ is water saturation of the whole core, fraction. $\Delta_{m}$ is water imbibed, ${\text{kg}}$. $L$ is sample length, m. $\rho_{w}$ is water density, ${\text{kg}}/{\text{m}}^{3}$.

In Eq. (8), the denominator represents the pore volume of the entire core.

Table 1 shows the maximum water saturation ${\text{S}}_{{{\text{wmax}}}}$ reached at the end of imbibition in the 12 specimens from the oil and gas reservoirs; the saturation ranged from 45 to 76%, and the mean saturation was 62.64%. According to traditional imbibition theories, all cores are in a position to be 100% saturated with water through imbibition. The experimental result is quite different from the theory, which reveals the complexities of the pore structures and the imbibition mechanisms of natural oil and gas reservoirs.

### Progresses characterization of imbibition front

**Figure 4:**
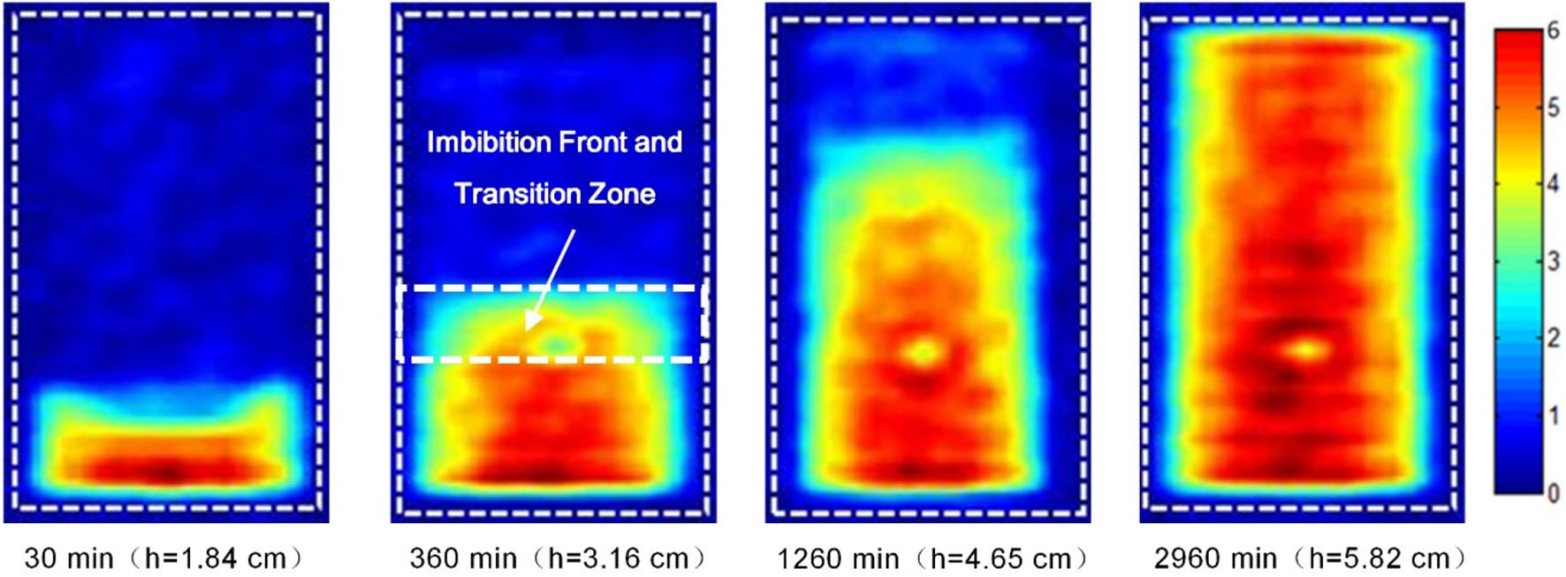
Images of water imbibition at different times.

Stratified NMR was used to determine the water saturation in sandstone specimen S6 at different times during the imbibition experiment (see Fig. 4). Because the measurement is indirect, colour columns 0–6 in the figure indicate the intensity of the water signal, which reflects the relative water saturation of the core; the larger the value, the greater the water saturation.

Figure 4 clearly illustrates the process by which the water phase is gradually sucked into the core as a wetting fluid during reservoir imbibition. First, there is a clear gas–water interface at the imbibition front, and the interface is essentially parallel to the air–water contact surface; with the passage of imbibition time, the interface gradually rises horizontally. Second, during imbibition, especially when the imbibition front does not reach the upper edge of the core, the water saturation value of the adjacent area behind the front edge is relatively low, and a distinct transition zone is visible (see the mark at 360 min in Fig. 4). In other words, the mechanism of imbibition in sandstone reservoirs is complex; it is not agree with a piston-like assumption. After the imbibition front reaches the upper edge of the core, the transition zone disappears along with the continual water phase fluid imbibition.

**Figure 5:**
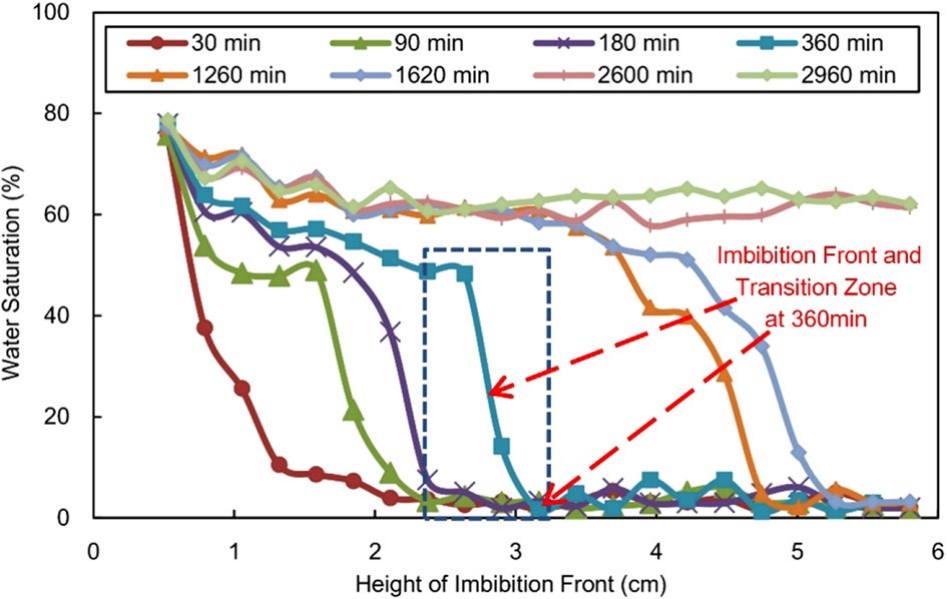
Distribution of water saturation at different times.

The water saturation in various segments of the core during imbibition is determined by converting the water saturation of the specimen based on the intensity of the NMR signal^45,46^ and through calibration using the weight method (Fig. 5). Figures 4 and 5 clearly depict the dynamic variation process of water saturation during reservoir imbibition: The greatest water saturation is observed at the gas–water contact surface (the first measuring point is 0.53 cm away from the end face), and the lowest saturation is observed at the imbibition front; there is an obvious transition zone (approximately 0.5–1.5 cm) behind the front edge; the water saturation increases sharply in the transition zone and remains noticeably unchanged behind the zone (see the mark at 360 min in Fig. 5); before the imbibition front reaches the upper edge of the core, the entire water saturation profile is approximately stepped.

The imbibition experiment reveals that the characteristics and variation patterns of the “transition zone” and the “residual gas” in the imbibition zone are vastly different from those in traditional theoretical hypotheses. According to the L-W imbibition theory, the gravity effect is negligible at the core scale, and all specimens should be completely saturated with water by imbibition. The experiment reveals the complexity of the pore structure and the imbibition mechanism of natural oil and gas reservoirs: On the one hand, due to pore heterogeneity, pore throats at different scales in reservoirs act as capillary bundles with various diameters, and the resulting differences in the capillary force lead to transition zone; on the other hand, the blind ends in the complex pore network formed by channels of different sizes and shapes and the “water-sealed gas” phenomenon caused by circumfluence, jamming, etc. during imbibition will inevitably result in the presence of residual gas, making the insufficient imbibition of the cores.

**Figure 6:**
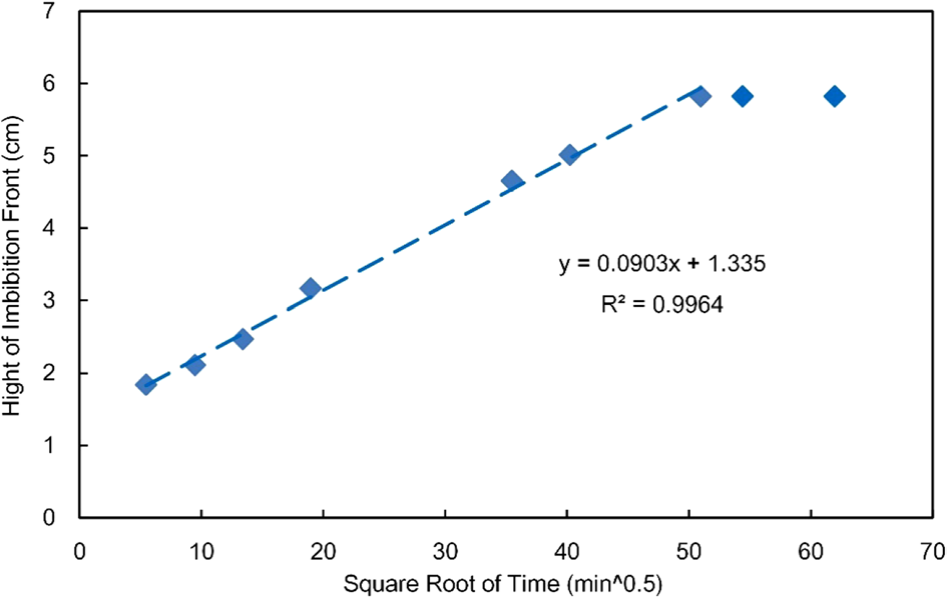
Hight of imbibition front versus the square root of time.

Figure 6 shows the statistics of the rising process of the imbibition front; with the upper boundary conditions in mind, within 2600 min (51, when converted to the square root of min), the ascent height has an excellent linear relationship with the square root of time when the front of specimen S6 does not reach the upper edge of the specimen.

9 $$h{ = 0}{\text{.0903}}\sqrt t + 1.335$$

Despite the different expressions of the rising height of the imbibition front in the L-W and Handy models, both models indicate that the advancement height/distance of the front is positively correlated with the square root of time. The experimental results substantially match the theoretical model results; however, the experimental fitting line does not pass through the origin, but intersects the upper half of the vertical axis. By all appearances, according to the theoretical model, the imbibition height should be 0 cm when the imbibition time is 0 min. The analysis suggests that the failure of the experimental fitting line to pass through the origin may be the synthetic result of experimental errors and the imbibition transition zone. First, due to the high imbibition rate at the beginning of the experiment, the test interval may cause measurement errors; moreover, to keep the lower end face of the core completely in contact with the water surface during the experiment, the lower end face is immersed in water (approximately 0.1 cm below level), which makes the initial boundary conditions of the experiment more conducive to imbibition. Second, the experiments demonstrate the presence of an imbibition front and a transition zone (that cannot be addressed by traditional theoretical models) in the intermediate reservoir, which may lead to high experimental values. The curve intercept (1.28 cm) in Fig. 6 is consistent with the range of the transition zone (0.5–1.5 cm) at different stages, which verifies the conclusion to a certain extent.

### Variation of water saturation

Known as a focus of the theory and experimental study of porous media imbibition, the variation of imbibed water quantity/water saturation in imbibition zone is particularly important for theoretical calculation (e.g., the Handy model). However, the above-noted NMR experiment demonstrates that the imbibition zone in the reservoir gradually grew larger during imbibition; at the early stage of imbibition, the actual range *h* of the imbibition zone was much smaller than the overall length $L$ of the specimen; traditional methods (as shown in Eq. (8), where the total weight method was used to take the mean water saturation of entire specimen as the water saturation of imbibition zone) will bring about dramatic deviation from reality, thereby leading to an incorrect understanding and conclusion.

Taking specimen S6 as an example, specimen length *L* should be replaced by the determined imbibition height *h* at each time point by NMR imaging to figure out the actual water saturation in the imbibition zone of specimen S6:

10 $$S_{w} = \frac{{\Delta_{m} }}{{\rho_{w} A\emptyset h}}$$

Table 2 shows the overall water saturation $S_{wt}$ of the core and the water saturation $S_{w}$ of the imbibition zone calculated at various time points during imbibition in S6 using Eqs. (8) and (10), respectively. By all appearances, $S_{w}$ will always be greater than $S_{wt}$ before the imbibition front reaches the upper edge of the core; the longer the specimen, the greater the difference. At the beginning of the present experiment (90–360 min, *h* $\ll L$), the difference exceeded 30%; but as $h$ gradually approached *L* in the later stage, the difference between $S_{w}$ and $S_{wt}$ decreased.

**Table 2:** The imbibition height and water saturation in the imbibition zone of sample S6.

Imbibition time, $t$/min	Height of imbibition front, $h$ /cm	Water imbibed, $\Delta_{m}$/g	Water saturation of sample, $S_{wt}$/%	Water saturation of imbibition zone, $S_{w}$/%
30	1.84	0.334	10.81	34.18
90	2.11	0.530	17.16	47.32
180	2.47	0.681	22.00	51.84
360	3.16	0.903	29.20	53.78
1260	4.65	1.379	44.64	55.87
1620	5.01	1.522	49.25	57.21
2600	5.82	1.858	60.09	60.09
2960	5.82	1.892	61.24	61.24
3840	5.82	1.968	63.68	63.68

**Figure 7:**
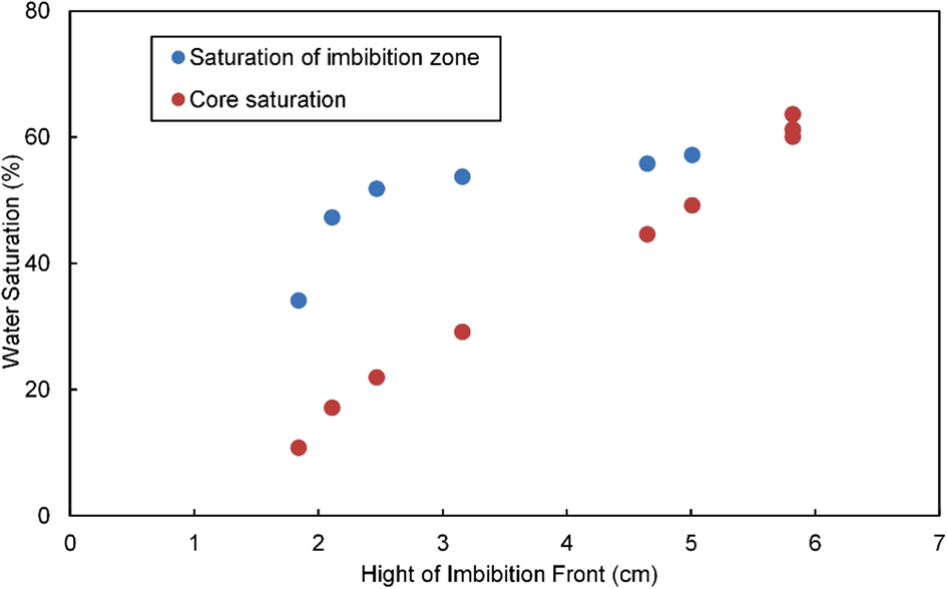
Dynamic water saturation with the expansion of the imbibition zone.

Figure 7 shows the dynamic variation in $S_{w}$ with the expansion of the imbibition zone during imbibition. With the expansion of the imbibition zone, the $S_{w}$ value in the imbibition zone increased and tended to be stable after the imbibition zone reached the upper edge of the core. The entire process was apparently composed of three stages: In the early stage (0–180 min, ${\text{h < 2}}{.5}$ cm), the $S_{w}$ value in the imbibition zone was low, but it increased rapidly and exceeded 50%; in the intermediate stage (180–2600 min, ${\text{h/L < 1}}$), which was the longest, the imbibition front progressively approached the upper edge of the specimen, while the growth rate of ${\text{S}}_{{\text{w}}}$ significantly decreased (only 8.3%); in the later stage (2600–3840 min, *h*/*L* = 1), the imbibition front reached the upper edge of the specimen, and the increase in the water content principally enhanced the transition zone saturation, while the $S_{w}$ value in the imbibition zone expanded at an extremely slow rate; at 3840 min, the transition zone disappeared completely, and the imbibition saturation reached the maximum value of 63.68% (the amplitude of the increase in $S_{w}$ was merely 3.6%).

Figures 4 and 5 show the dramatic stepped change in the water saturation of the transition zone, while the saturation behind the transition zone remained substantially stable. Accordingly, the analysis results suggest that the presence of the transition zone is the main cause of the variation of $S_{w}$ in the imbibition zone, as shown in Fig. 7. In the early stage, the transition zone accounted for a high proportion of the imbibition zone, and the $S_{w}$ value in the imbibition zone was significantly affected by the transition zone (varying within wide limits); along with the fluid imbibition, the imbibition zone expanded and the proportion of the transition zone decreased; its effect weakened, while the variation in $S_{w}$ in the imbibition zone slowed down; after the imbibition front reached the upper edge of the core in the later stage, the transition zone faded away, while the $S_{w}$ value of the imbibition zone tended to be a fixed value.

## Model optimization and verification

Studies have shown that the process and mechanism of imbibition are more complicated in oil and gas reservoirs and that the hypothesis and simplification methods of the L-W and Handy models inevitably introduce errors. Therefore, it is necessary to verify the applicability of the traditional theoretical models through experiments and to correct the calculated values, thereby improving the applicability and accuracy of the models for oil and gas reservoirs.

### Optimization and correction of the L-W model

According to the preceding context, oil and gas reservoirs feature a complex pore structure, so accurately measuring r is difficult, which restricts application of the L-W model. Thus, the L-W model was optimized based on the theory of fluid mechanics.

According to the Poiseuille's Law and the Darcy-Weisbach Formula, the relationship between the mean channel radius and the permeability of a reservoir has been established^47,48^:

11 $$r{ = }\tau \sqrt {\frac{{8K_{a} }}{\emptyset }}$$

where $\tau$ is tortuosity of pore. $K_{a}$ is gas permeability, ${\text{m}}^{2}$.

By substituting Eq. (3) from front rising height of the L-W equation, the following equation is obtained:

12 $${\text{h = }}\left( {\frac{{2K_{a} }}{\emptyset }} \right)^{{{\raise0.7ex\hbox{$1$} \!\mathord{\left/ {\vphantom {1 4}}\right.\kern-\nulldelimiterspace} \!\lower0.7ex\hbox{$4$}}}} \cdot \sqrt {\frac{{\tau \sigma {\text{cos}}\theta }}{\mu }t}$$

According to the analysis above, the water saturation of the imbibition zone is a variable, and there is a transition zone, so it is difficult to calculate the imbibition amount directly from the imbibition height. In practice, the effect of the transition zone can be neglected when the imbibition zone is far greater than the transition zone. With reference to previous studies, to simplify the calculation, the maximum water saturation ${\text{S}}_{{{\text{wmax}}}}$ at the end of core the experiment replaces the mean saturation $S_{w}$ when calculating the imbibed water quantity of a reservoir. The imbibed water quantity in the core can be expressed as follows:

13 $$W_{L - w} = \rho AS_{{{\text{wmax}}}} \emptyset h = \rho AS_{{{\text{wmax}}}} \cdot \left( {2K_{a} \emptyset^{3} } \right)^{1/4} \sqrt {\frac{\tau \sigma \cos \theta }{\mu }t}$$

Experiments have indicated that the transition zone has a great impact in the early stage, and the mean water saturation $S_{w}$ of a specimen is much lower than ${\text{S}}_{{{\text{wmax}}}}$; in the later stage, the imbibition zone expands, and the transition zone has a less significant effect, while the mean water saturation $S_{w}$ of the specimen gradually approaches ${\text{S}}_{{{\text{wmax}}}}$.

Equations (12) and (13) are optimized L-W equation expressions.

The fluid factor is defined as follows:

14 $$F{ = }\rho \sqrt {\frac{\sigma \cos \theta }{\mu }}$$

According to dimensional analysis, the unit of *F* should be ${\text{kg}}/({\text{m}}^{5} \cdot {\text{s}})^{1/2}$.

The reservoir factor is defined as follows:

15 $$R{\text{ = A}}\left( {2K_{a} \emptyset^{3} \tau^{2} } \right)^{1/4}$$

According to dimensional analysis, the unit of $R$ should be ${\text{m}}^{5/2}$.

Then, the imbibed water quantity in the core can be expressed as follows:

16 $$W_{L - w} = FR \cdot {\text{S}}_{{{\text{wmax}}}} \sqrt t$$

where ${\text{S}}_{{{\text{wmax}}}}$ is the maximum water saturation at the end of the experiment, fraction.

Parameter groups $F$ and $R$ represent the principal factors affecting the reservoir imbibition effect. According to the definition, fluid factor $F$ principally characterizes the properties of the imbibition fluid, including the fluid density, the viscosity, the interfacial tensions, which are tightly associated with the imbibition power and the capillary force, and the fluid contact angle; reservoir factor $R$ principally characterizes the properties of the rock reservoir that act as imbibition and seepage channels, including the contact area $A$ between the reservoir and the fluid, as well as the macroscopic physical parameters such as the porosity $\emptyset$, permeability $K_{a}$, and tortuosity $\tau$ of the microscopic physical parameter channels. Fluid factor $F$ and reservoir factor $R$ are derived by strict theoretical derivation; as a result, they have a clear physical significance and are easy to accurately test. After $F$ and $R$ have been defined, it is easy to determine imbibition height/distance and the imbibition amount.

Under normal circumstances, the imbibition fluid media is given, and the fluid factor $F$ is a fixed value; this, it is only necessary to determine the reservoir factor $R$ based on the reservoir parameters. Under the experimental conditions, the gas–water interfacial tension is $\sigma {\text{cos}}\theta = 72 \times 10^{ - 3} {\text{N}}/{\text{m}}$, the water phase density is $\rho = 1 \times 10^{3} {\text{kg}}/{\text{m}}^{3}$, and the viscosity is $\mu = 1.005 \times 10^{ - 3} {\text{Pa}} \cdot {\text{s}}$; thus, the fluid factor $F = 8464.15{\text{kg}}/({\text{m}}^{5} \cdot {\text{s}})^{1/2}$.

**Figure 8:**
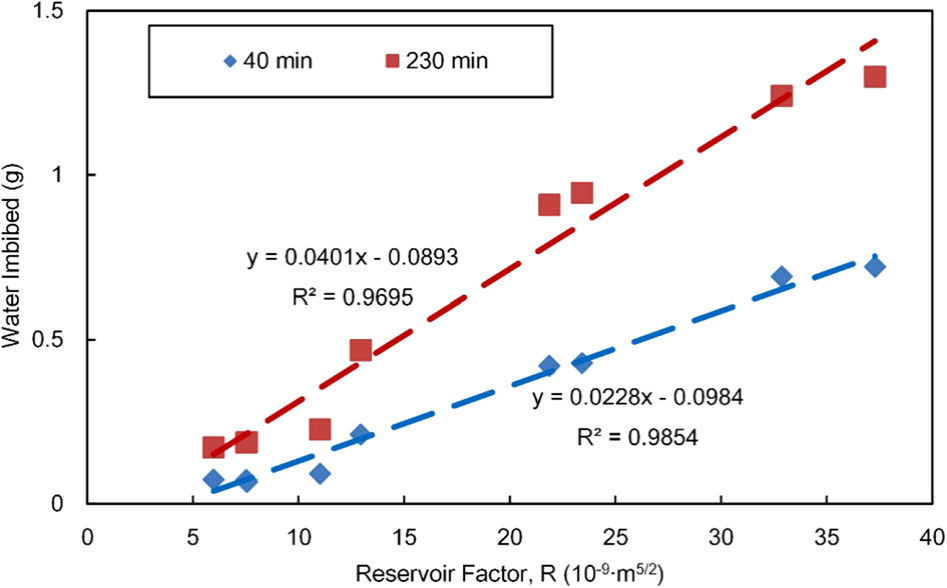
Water imbibition versus the reservoir factor R.

According to previous research findings, the tortuosity of natural sandstone reservoirs has an empirical value of τ = 2^48,49^, and all physical parameters of a specimen should be expressed in international units; thus, the reservoir factors $R$ of various specimens can be calculated, as shown in Table 1. During the experiment, the fluid factor F is a constant value, and the reservoir imbibition capacity and imbibed water quantity are only associated with the reservoir factor $R$. Figure 8 shows the relationship between the measured imbibition amount and the reservoir factor $R$. The imbibed water quantity of a specimen at each time point (40 min, 230 min) is linearly related with the reservoir factor $R$, where the correlation coefficient is larger than 0.96. From this point of view, the reservoir factor $R$ and the optimized L-W model can favourably characterize the effect of the reservoir and the fluid on the imbibition amount and thus have an excellent guiding significance for defining the reservoir imbibition distance and scale.

**Figure 9:**
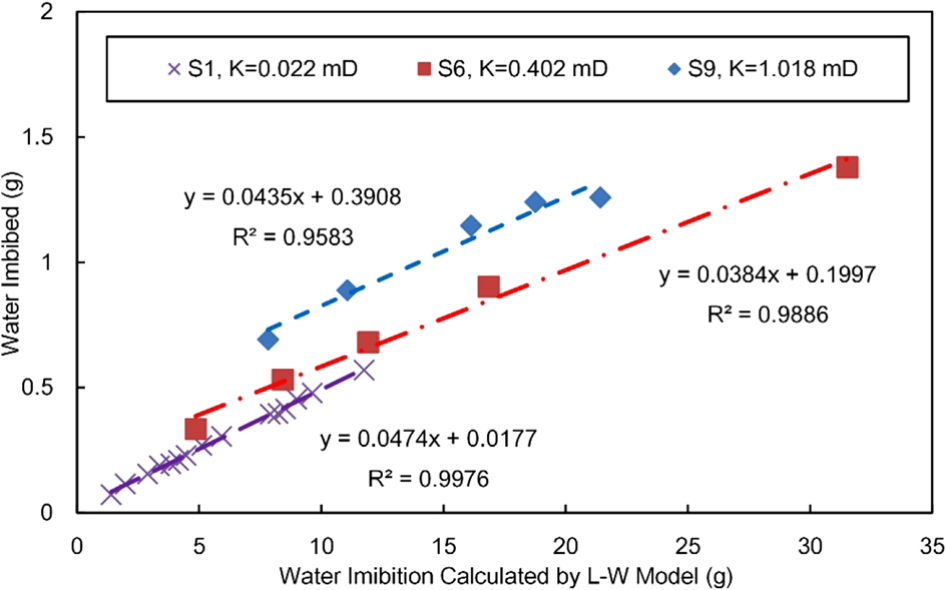
Experimental water imbibition versus the calculated value of L-W model.

As discussed above, the assumption of L-W model is quite different from the actual imbibition process of reservoir. The main parameters of the model have a good correlation with water imbibition, which does not mean that the theoretical calculation is accurate. The application of the model in reservoirs needs to be modified by experiments. Figure 9 is the curves between the experimental water imbibition and the calculated value of L-W optimization model (Eq. (13)).The comparison shows that: on the one hand, for the three typical samples with large permeability span, there is a big gap between the calculated and measured values of the imbibition water weight, and the theoretical value is 20–30 times larger than the measured value; On the other hand, the correlation between the calculated results of the model and the measured results is very good, which indicates that the optimized model can accurately predict the imbibition weight and hight of reservoirs after proper correction.

Figure 9 shows that the linear equation does not pass through the origin, which results from the imbibition front and the transition zone according to the above analysis. With this intercept neglected, fitting is performed directly with the correction constant:

17 $$W_{{{\text{measured}}}} { = }C_{1} \cdot W_{L - w} = C_{1} \cdot FR \cdot {\text{S}}_{{{\text{wmax}}}} \sqrt t$$

where $C_{1}$ is the empirical correction coefficient of the optimized L-W model in gas–water imbibition in oil and gas reservoirs, and its value is approximately between 0.038 and 0.048. The value range of $C_{1}$ is not wide for sandstone reservoirs, which indicates that the correction coefficient is well defined; hence, the theoretical formula of the imbibition amount can be used after simple correction.

It can be inferred from Eq. (13) that the front rising height correction formula for oil and gas reservoirs is

18 $$h_{{{\text{measured}}}} = C_{1} FR \cdot {{\sqrt t } \mathord{\left/ {\vphantom {{\sqrt t } {\left( {\rho A\phi } \right)}}} \right. \kern-\nulldelimiterspace} {\left( {\rho A\phi } \right)}}$$

It should be noted that because measuring the imbibition height is difficult, it is a common practice to obtain a quality correction formula (Eq. (17)) by experimental fitting and to thereby infer the imbibition front height correction formula (Eq. (18)). However, the experimental and derivation processes clearly showed that the tremendous difference between the theoretical imbibition height and the measurements was the principal factor leading to the difference in the imbibition amount (the water saturation $S_{w}$ value did not cause large errors).

### Correction of Handy model

The application of Handy model needs to measure the water saturation ($S_{w}$) in the imbibition area, the capillary force of the reservoir ($P_{c}$) and the effective permeability of water ($K_{w}$) at this water saturation ($S_{w}$). However, capillary force ($P_{c}$) and water permeability ($K_{w}$) are very sensitive to water saturation ($S_{w}$). The measurement of these two sensitive parameters increases the difficulty and uncertainty of theoretical calculation.

As mentioned above, Handy et al. assumed the water saturation in the imbibition area was a constant value. According to the measurement standards of capillary force curve (GB/T 29171-2012)and gas water relative permeability curve(GB/T 28912-2012) , the $S_{w}$, $P_{c}$ and $K_{w}$ at the end of the imbibition experiments were measured. The results are shown in Table 3.

**Table 3:** Water permeability and capillary force of samples.

Sample numbers	Porosity, $\emptyset$/%	Gas permeability, $K_{a}$/mD	Water saturation in imbibition zone, ${\text{S}}_{{{\text{wmax}}}}$/%	Water permeability, *K*_*w*_/mD	Capillary force, $P_{c}$*/*MPa
S1	6.74	0.022	45.05	0.003	1.172
S6	10.74	0.402	61.24	0.072	0.310
S9	13.37	1.018	57.39	0.255	0.076

**Figure 10:**
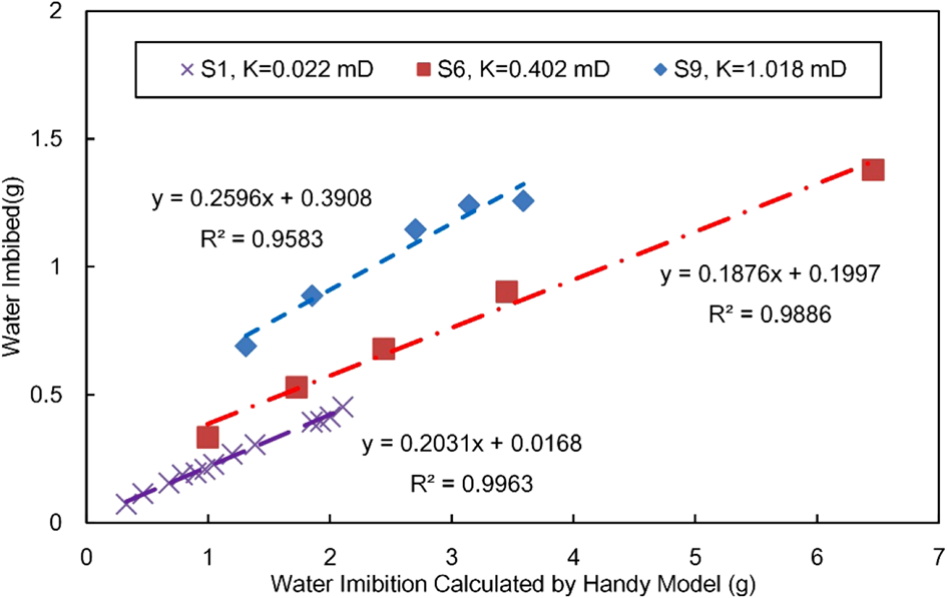
Experimental water imbibition versus the calculated value of Handy model.

The measured water imbibition and the predicted values of Handy model (Eq. (7)) are shown in Fig. 10 Similar to the results of L-W model, the theoretical values are different from the measured, but the correlation of them are strong. The correlation coefficient is above 0.95. The predicted value of handy model is also different from the measured value, and it is about 3–6 times of the measured.

Ignoring the intercept, the modified imbibition water of handy model is as follows:

19 $$W_{{{\text{measured}}}} { = }C_{2} \cdot W_{H} = C_{2} \cdot \rho A\sqrt {\frac{{2P_{c} K_{w} \emptyset {\text{S}}_{{{\text{wmax}}}} t}}{{\mu_{w} }}}$$

where $C_{2}$ is the empirical correction coefficient of Handy model in the process of gas water imbibition. According to the experimental results, the range of $C_{2}$ is about 0.18–0.26.

According to Eq. (5), the correction formula of front height of Handy model is as follows:

20 $$h = C_{2} \cdot \sqrt {\frac{{2P_{c} K_{w} }}{{\emptyset {\text{S}}_{{{\text{wmax}}}} \mu_{w} }} t}$$

### Analysis and discussion

The results show that although the predicted value of L-W optimization model and Handy model have a large deviation from the experimental value, the predicted value has a strong correlation with the measured. The applicability and accuracy of the two models in reservoirs are greatly improved after simple correction by correction coefficients $C_{1}$ and $C_{2}$.At the same time, because the values range of $C_{1}$ and $C_{2}$ are relatively concentrated, the models have strong operability in engineering application. It is a practical method to verify and modify the traditional model by experiment, before the perfect and accurate theoretical model is established.

In the L-W optimization model, the main parameters, fluid factor *F* and reservoir factor $R$ have clear physical meaning, which are easy to test accurately, and can better describe the variation of imbibition weight and distance. The model can be well applied to oil and gas reservoirs after experimental modification. The calculated values of handy model are closer to the measured values ($C_{2}$ is closer to 1), but the model needs to test more parameters ($P_{c}$ and $K_{w}$), and the related parameters are more difficult to accurately test.

Therefore, the L-W optimization model is more practical than the Handy model on the premise that both of them need to be modified by experiments and the accuracy of them is similar.

## Conclusions

The adaptability of traditional theoretical models of imbibition to oil and gas reservoirs was analysed. Experimental studies on gas–water imbibition in a reservoir were carried out on this basis. The experimental results show that the maximum saturation of water phase imbibition in oil and gas reservoirs was between 45 and 76% (mean: 62.64%); contrary to the theoretical hypothesis, water imbibition was a non-piston-like process, and there was a clear transition zone at the imbibition front; the transition zone is the main cause of variation in the water saturation in the imbibition zone; with the expansion of the imbibition zone, the influence of the transition zone on water saturation weakens; traditional theoretical models predict a strong correlation between the predicted quantity of imbibed water and measurements, but deviations exist in the numerical values, so corrections must be made.

The optimized L-W model was derived and then verified and corrected through experiments. The optimized and corrected model can favourably describe the variation of the distance and amount of imbibition. This model exhibits excellent engineering applicability and is thus suitable for oil and gas reservoirs.

The optimization models and correction methods regarding the gas phase and water phase in oil and gas reservoirs are also applicable for describing the oil–gas and oil–water imbibition effects. In studies and applications, it is necessary to use appropriate imbibition experiments and achieve corresponding correction coefficients depending on the actual imbibition fluid.
